# Stochastic simulations to optimize genomic selection for laying hens: Impact of generation interval and genotyping in the context of extended laying period

**DOI:** 10.1016/j.psj.2026.106870

**Published:** 2026-03-27

**Authors:** Mathilde Doublet, Frédéric Lecerf, Torsten Pook, Hervé Chapuis, Jérôme Raoul, Florian Herry, Sophie Brad-Fudulea, Gwendal Restoux, Florence Phocas, Sophie Allais

**Affiliations:** a PEGASE, INRAE, Institut Agro, Saint Gilles 35590, France; b Université Paris-Saclay, INRAE, AgroParisTech, GABI, Jouy-en-Josas, France; c SYSAAF, Station LPGP/INRAE, Campus de Beaulieu, Rennes 35042, France; d GenPhySE, Université de Toulouse, INRAE, Castanet-Tolosan, France; e Institut de l’Elevage, Castanet-Tolosan, France; f Wageningen University & Research, Animal Breeding and Genomics Group, Wageningen 6708 PB, the Netherlands; g Grimaud Frères Sélection, Sèvremoine 49450, France

**Keywords:** Laying hens, Genomic selection, Generation interval, Inbreeding, Stochastic simulations

## Abstract

Extending the productive lifespan of laying hens is a key objective for sustainable egg production. Achieving this goal requires improving egg production and quality traits expressed late in life. Genomic selection offers opportunities to increase accuracy of selection for such traits, while shorter generation intervals can accelerate genetic progress. However, both strategies may affect inbreeding, and their combined impact in the context of extended laying cycles has not yet been quantified. Stochastic simulations were performed to evaluate seven breeding programs for layers, based on real genotype data and six quantitative traits (egg weight, egg shell strength, and laying rate, each at 60 and 90 weeks). Programs differed by generation interval (*L*, 60, 45, or 30 weeks) and two selection method were applied to each scenario: single-step GBLUP using male genotypes (ssGBLUPm), or single-step GBLUP using both male and female genotypes (ssGBLUPmf). A control PBLUP based scenario with a 60 weeks *L* was also performed. Each scenario was replicated 30 times, and results were compared for annual genetic gain (∆G), prediction accuracy (*r*), and inbreeding rate (∆F). Genomic evaluations using a generation interval of 60 weeks improved both ∆G and ∆F, especially when both sexes were genotyped. Reducing the generation interval to 30 weeks maximized ∆G (up to 1.17 SD/year) but increased ∆F above 1%/year. Overlapping generation schemes (45-week interval) provided an intermediate outcome, improving ∆G compared with conventional 60-week generation interval schemes while limiting ∆F compared with 30-week generation interval schemes. Including female genotypes was particularly beneficial for late-recorded traits at 90 weeks, where accuracy increased by up to 38%. Shortening generation interval and implementing genomic selection substantially increased annual genetic gain, especially for persistency traits expressed late in life. However, these strategies also raised inbreeding, with overlapping generations offering a valuable compromise. Full genotyping of both sexes enhanced accuracy and reduced the increase in ΔF per ΔG unit, highlighting the relevance of genomic selection in breeding programs aiming to extend laying periods sustainably.

## Inttroduction

Laying persistence is becoming increasingly important as the industry seeks to extend the productive lifespan of hens beyond 90-100 weeks, for both economic and environmental reasons (Bain et al., 2016; Preisinger, 2018). Currently, the main reason for culling hens around 75 weeks of age is the decline in egg number and the deterioration of eggshell quality with advancing age (Bain et al., 2016). Consequently, breeding programs must aim to select hens capable of maintaining high egg quality and production at older ages, thereby achieving genetic gain for these late-life traits in addition to conventionally recorded ones. However, selecting for such traits is not straightforward. Phenotypes measured during the conventional laying period may not be sufficient, as the genetic architecture of traits evolves over time. Genetic variances are known to change with age (Engström et al., 1992), and heritability estimates of quantitative traits in laying hens are similarly age-dependent. For example, the heritability of eggshell traits tends to decline with age due to increased residual variance (Liu et al., 2021; Ni et al., 2023). Moreover, genetic correlations between production traits such as egg number or the number of clutches until cx weeks of age, measured at two periods of the life are generally moderate to high typically ranging from 0.5 to 0.9 (Liu et al., 2019; Ni et al., 2023). Studies have shown that correlations between the same trait recorded at different age are strongly positive but statistically different from

1 also for egg quality traits (Liu et al., 2018). These moderate to high genetic correlations suggest that early-life performance provides some indication about late-life performance, but does not fully capture the genetic potential for persistency. Therefore, laying persistency traits should be considered as independent selection objectives in breeding programs to fully exploit their genetic potential and improve their selection efficiency.

The evaluation of persistency traits is challenging, as they are expressed late in life and therefore costly and time-consuming to record. Therefore, selection candidates (and even highly related animals) are rarely phenotyped for these traits at timepoint of selection. **Genomic Selection (GS)** offers a promising approach for evaluating such traits, as it enables accurate prediction of breeding values based solely on genotypic information, even in the absence of individual phenotypic records. GS relies on dense molecular markers, such as **single nucleotide polymorphisms (SNPs)** to derive **genomic estimated breeding values (GEBVs)** by exploiting the associations between genotypes and phenotypes in a reference population. Among the various GS models, those based on Henderson’s **Best Linear Unbiased Prediction (BLUP)** equations (Henderson, 1975) are the most widely used. In Meuwissen et al. (2001) proposed a model where the pedigree-based relationship matrix is replaced by a genomic relationship matrix that accounts for the Mendelian sampling. In 2010, a **single-step GBLUP (ssGBLUP)** method, which integrates both genomic and pedigree information into a unified model was proposed (Aguilar et al., 2010; Christensen and Lund, 2010). The ssGBLUP model enables the full use of genotypic and phenotypic information of the population to improve the **accuracy (*r*)** of breeding value prediction, particularly for individuals without own phenotypes (Nejati-Javaremi et al., 1997; Villanueva et al., 2005; Hayes et al., 2009). Later in Legarra et al. (2014) proposed easy to implement equations for ssGBLUP. In laying hens, many economically important traits are sex-limited or late-recorded. For instance, egg quality traits and reproduction traits are recorded only in females, while other traits, such as laying persistence, are unavailable at the time of selection. In these cases, genomic selection improves *r* of the evaluation compared to traditional pedigree-based approaches (Wolc et al., 2011b; Lourenco et al., 2015).

An increase in *r* is desirable in animal breeding, as it is linearly correlated with **annual genetic gain (ΔG)** (Falconer, 1989). Although generally higher than the accuracy of pedigree-based selection, the accuracy of GS remains variable, and, as in traditional genetic evaluations, depends on population structure. The accuracy increases when candidates are closely related to individuals in the reference population (Habier et al., 2010; Clark et al., 2012; Pszczola et al., 2012). In addition, reference population size has an important positive effect (Wolc et al., 2011a; Weng et al., 2016). Trait-specific parameters, such as heritability, play also a role. Marker characteristics, number, density, distribution, and genotyping quality, also matter (Herry, 2019). Since the gain in *r* from GS is context-dependent, breeding programs must be optimized to ensure that their improvement effectively translates into additional genetic gain.

Moreover, genomic information can help to reduce the **generation interval (*L)***. A shorter *L* increases annual ΔG by enabling more selection cycles within a given timeframe. This strategy is widely applied in livestock breeding programs, including species that already have a short *L*, such as laying hens (Wolc et al., 2016). However, reducing *L* introduces a trade-off with *r*. Early selection based on **estimated breeding values (EBVs)** before phenotypes are available for the candidates or their contemporary relatives, can reduce *r* compared to EBVs estimated when phenotypes are recorded on selection candidates or their relatives. This statement is particularly true for late-measured traits as the prediction accuracy of EBVs decreases while the genetic distance between phenotyped and candidate individual decreases. In many cases, the gain in *r* from GS compensates for this loss (Sitzenstock et al., 2013; Wolc et al., 2016). As a result, combining GS with a reduced *L* often results to higher ΔG over time.

Still, the trade-off between *r* and *L*, and its effect on ΔG, remains rarely quantified. Moreover, modifying *r* and *L* can also affect the **annual inbreeding rate (ΔF)**. Since ΔF constrains long-term genetic gain and population sustainability, it should be explicitly considered when optimizing breeding programs.

Stochastic simulation of breeding programs allows comparison of alternative breeding strategies prior to implementation. In simulations, a digital twin of the breeding program is generated that allows to assess the impact of concrete changes to the breeding program. It offers high flexibility and opportunities for in-depth and detailed analysis of breeding programs strategies. Previous simulation studies have shown the benefits of GS for laying hens for conventional laying period, with a *L* reduction (Sitzenstock et al., 2013; Büttgen et al., 2025). However, its potential has not yet been evaluated for breeding programs aiming to lengthen laying periods for hens while implementing GS features.

Therefore, the objective of this study was to investigate the balance between *r* and *L* on ΔG in laying hen breeding programs, while accounting for the annual ΔF. The programs were designed to include both production and egg quality traits measured up to 90 weeks, in line with the industry's goal of improving laying persistence. Six selection programs differing in their generation interval and number of genotyped individuals in the population were simulated to be compared with each other, as well as with a baseline pedigree-based breeding program.

## Materials and methods

### Simulation initialization

**Fig. 1:**
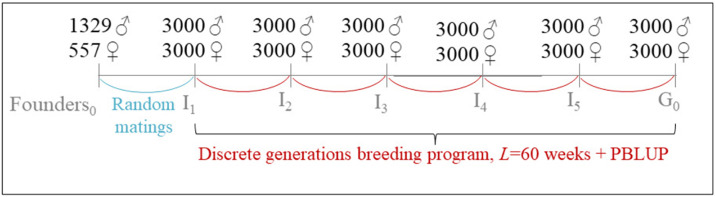
**Initialization of the breeding program.** Individuals from the Founders₀ population were randomly mated to produce generation I₁. A discrete generation breeding program was then applied over five generations (I₁ to I₅), with a generation interval of 60 weeks and random matings avoiding half and full sibling mating. During this burn-in phase, selection was based on pedigree-based BLUP (PBLUP).

To simulate a realistic genetic background, real genotype data provided by a breeding company (Novogen, Plédran, France) were used to construct the base population. This founder population, referred to as Founders_0_, consisted of 557 females and 1329 males (Fig. 1). No pedigree information was available for Founders_0_. Genotyping was performed using a custom 60 K SNP chip derived from the Affymetrix® Axiom® 600 K SNP array developed by Kranis et al. (2013). A total of 42,305 SNP markers evenly distributed across the genome were retained after quality to simulate the genome. To model recombination in meiosis, genetic position in Morgan were derived by converting physical position with a fixed ratio of 30,000,000 base pairs per Morgan.

### Trait simulation

Six quantitative traits were simulated. They correspond to the **egg weight (EW)**, the **egg shell strength (ESS)** and the **laying rate (LR)**, measured at two different laying phases. The EW was measured in grams. The ESS corresponded to the strength in Newton applied to break the shell. The LR was calculated as the total number of eggs produced dived by the period duration and multiplied by 100 (i.e. as percentage). Those 3 traits were simulated for 2 periods of the laying period. From 30 to 60 weeks of age (EW_60_, ESS_60_, LR_60_), the traits were referred to as 60-week traits. That time corresponds to the laying peak production period. From 60 to 90 week of age (EW_90_, ESS_90_, LR_90_) corresponding to the laying persistency period, the traits were referred to as 90-week traits. The six traits assumed 1000 underlying purely additive quantitative trait loci for each, with effect sizes drawn from a Gaussian distribution and heritability of $h_{EW_{60}}^{2}=0.6$, $h_{EW_{90}}^{2}=0.45$, $h_{ESS_{60}}^{2}=0.25$, $h_{ESS_{90}}^{2}=0.25$, $h_{LR_{60}}^{2}=0.20$ and $h_{LR_{90}}^{2}=0.20$ respectively (Table 1). Heritability values were fixed based on expertise (literature review and personal communications with breeders). Traits were standardized to have a true genetic mean value of 0 and a genetic variance of 1. Correlations between the same trait measured at different times (60 weeks and 90 weeks) were set to 0.7. The correlations between two different traits measured at two different times (for example EW_60_ and ESS_90_) were set as the average between the correlations of these traits when they were measured at the same time (the mean between $cor_{EW_{60}/ESS_{60}}$ and $cor_{EW_{90}/ESS_{90}}$) multiplied by 0.7, according to the correlation tested scenario across time (Table 1).

**Table 1:** Genetic parameters for the 6 traits using 0.7 as the correlation between two traits measured at different ages. Heritabilities on the diagonal and genetic correlations above the diagonal. EW: egg weight, ESS: egg shell strength, LR: laying rate.

	*EW_60_*	*ESS_60_*	*LR_60_*	*EW_90_*	*ESS_90_*	*LR_90_*
**EW_60_**	0.60	−0.07	−0.11	0.70	−0.07	−0.11
**ESS_60_**		0.25	−0.19	−0.07	0.70	−0.19
**LR_60_**			0.20	−0.11	−0.19	0.70
**EW_90_**				0.45	−0.07	−0.11
**ESS_90_**					0.25	−0.19
**LR_90_**						0.20

### General structure of the breeding programs

The initial population of 3000 males and 3000 females was generated in two successive burn-in phases to ensure a realistic population structure and a pedigree for ssGBLUP. First, random mating among Founders_0_ was simulated to generate an initial generation I_1_ composed of 3000 males and 3000 females. Starting from I_1_, a breeding program with discrete generations and a *L* of 60 weeks was simulated for five generations. In second burn-in phase, genetic evaluations were performed using **pedigree-based BLUP (PBLUP)**. Selected males were randomly mated with 8 different females to ensure the birth of 10 offspring per female. Mating between half or full siblings was avoided. The final generation, obtained by mating I_5_ males and females, constituted generation G_0_ (Fig. 1). This generation served as the starting point for all selection scenarios evaluated in this study.

**Fig. 2:**
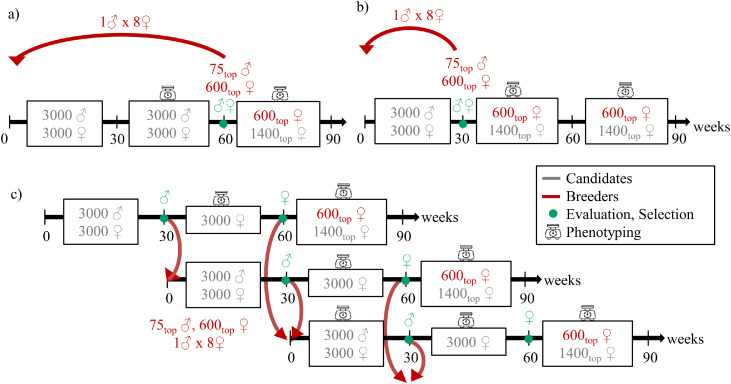
**Description of the three types of breeding programs.** a) breeding programs with discrete generations and a 60-week generation interval. b) breeding programs with discrete generations and a 30-week generation interval. c) breeding programs with overlapping generations and a 45-week generation interval.

The burn-in was followed by a 600 weeks evaluation period of three types of breeding programs. The first type assumed discrete generations with *L* of 60 weeks (Fig. 2a). Per generation, 3000 females were phenotyped for the 60-week traits and the 2000 top, based on their EBVs, were phenotyped for the 90-week traits. A second type of breeding program assumed discrete generations with a *L* of 30 weeks (Fig. 2b). Per generation, 2000 females were phenotyped for the 60-week and the 90-week traits. In these two types of programs, all parents were replaced at each cycle, and matings occurred strictly within cohorts. The third type of breeding program assumed overlapping generations in which only the male *L* was reduced to 30 weeks, while the female *L* remained at 60 weeks so they could benefit from their own performances in the evaluations. This led to an average *L* of 45 weeks (Fig. 2c). Per generation, 3000 females were phenotyped for the 60-week traits and 2000 were phenotyped for the 90-week traits.

### Estimate of breeding value

*S*election was based on breeding values that were estimated using BLUPf90 (Misztal et al., 2002). For each genomic breeding program, a multi-trait ssGBLUP combining the 6 selected traits was used for the breeding value estimation. In scenarios where only male candidates were genotyped, the evaluation was referred to as “ssGBLUPm.” When both male and female candidates were genotyped, the evaluation was referred to as “ssGBLUPmf.” To reduce the computational demand, the genotypes of non-breeding individuals from the reference population were excluded from the model. In breeding programs with discrete generations and a *L* of 60 weeks, a third evaluation method, pedigree-based BLUP (PBLUP), was also tested. This approach relied solely on phenotypic and pedigree information for EBV estimation. In all breeding value estimations, phenotypic records from the last four generations were used, as recommended by Weng et al. (2016). A synthetic index was derived by combining the six (G)EBVs. The weights for EW_60_, ESS_60_, LR_60_, EW_90_, ESS_90_ and LR_90_ were respectively 0.06, 0.16, 0.22, 0.06, 0.22, 0.28. They were chosen empirically to focus on the ESS and LR and particularly on the persistency traits because ESS and LR tend to deteriorate with time (Bain et al., 2016). Evaluations were carried using the underlying true heritabilities and genetic correlations derived based on underlying true genomic values and residual effects to save computing time. The last four generations, including the candidate one, were included in the evaluations. The causal variants were excluded from the marker set used in the ssGBLUP evaluations to avoid artificially inflating prediction accuracy.

The top 75 males and 600 females were selected as parents of the next generation. Each selected male was randomly mated with 8 of the selected females, and each mating produced a total of 10 offspring per female. To reduce the risk of inbreeding, full-sib, half-sib, and parent-offspring matings was avoided. The top 2000 females including the 600 selected breeders, were reared until 90 weeks of age. A mortality rate of 3% was applied to all selection candidates, based on observed mortality rates under individual cage conditions (Bécot, 2023).

**Fig. 3:**
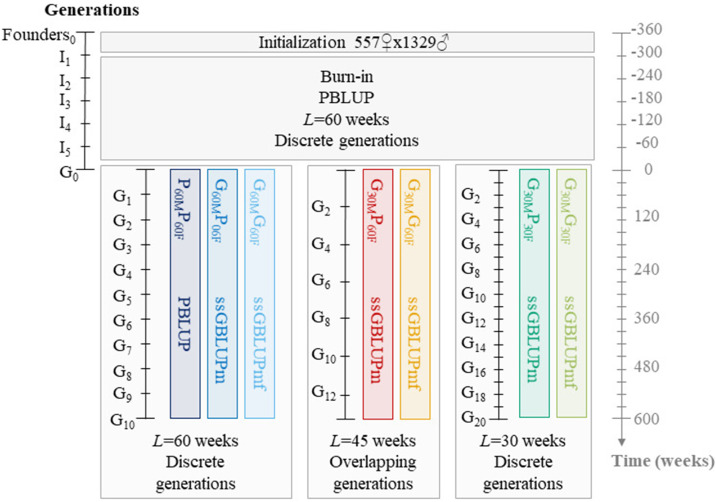
Overview of breeding program progression from founders to 600 weeks, including burn-in phase.

Three 60-week *L* breeding programs were tested: PBLUP (P_60M_P_60F_), ssGBLUPm (G_60M_P_60F_), and ssGBLUPmf (G_60M_G_60F_). For the 45-week *L* program with overlapping generations, two strategies were evaluated: ssGBLUPm (G_30M_P_60F_) and ssGBLUPmf (G_30M_G_60F_). For the 30-week *L* program with discrete generations, the same strategies were applied: ssGBLUPm (G_30M_P_30F_) and ssGBLUPmf (G_30M_G_30F_). Fig. 3 and Table 2 summarize all scenarios, and schematic representations are provided in Additional Figure S1. All simulations were carried using under R version 4.4.0 using the package MoBPS in the version 1.11.82 (Pook et al., 2020).

**Table 2:** Material used for the evaluation of candidates for each cohort, per scenario.

	*Number of phenotyped females per cohort*		*Number of individuals genotyped in the reference population*		*Type of BLUP*
	60	90	M	F	
P_60M_P_60F_	3000	2000	-	-	PBLUP
G_60M_P_60F_	3000	2000	225	-	ssGBLUPm
G_60M_G_60F_	3000	2000	225	900	ssGBLUPmf
G_30M_P_60F_	3000	2000	225	-	ssGBLUPm
G_30M_G_60F_	3000	2000	225	900	ssGBLUPmf
G_30M_P_30F_	2000	2000	225	-	ssGBLUPm
G_30M_G_30F_	2000	2000	225	900	ssGBLUPmf

### Comparison of breeding programs

The simulations were replicated 30 independent times to assess variability in the results. This number of replicates was increased compared to comparable studies (Pocrnic et al., 2023; Büttgen et al., 2025). Results were processed with R version 4.4.0. The breeding scenarios were compared based on three criteria: (i) the mean accuracy of the evaluations, (ii) the mean genetic gain (ΔG) and (iii) the mean inbreeding rate (ΔF). The accuracy of the evaluations was calculated for each candidate cohorts of each scenario as the average correlation between the EBVs and the **true breeding values (TBVs)** across the 30 replicates. The *r* values were calculated at a population level, distinctly for each trait. It was also calculated distinctly for males and females. The differences in*r* between the 7 scenarios were tested with a t-test (Student, 1908).

The cumulated ΔG for each elementary trait was defined as the difference of TBV values expressed in genetic standard deviation between the population from the **initial generation (G_0_)** resulting from the burn-in and the population from the last generation, after 600 weeks of selection. It was scaled by the mean and standard deviation values at generation G_0_. The final values of cumulated ΔG represent averages across the 30 replicates. The annual ΔG was the cumulated ΔG divided by 11.5 years.

The cumulated ΔG gain was also measured on the synthetic breeding goal (H), i.e. the combination of TBV combining the 6 traits. The mean synthetic breeding goal was calculated as the linear combination of the mean TBV values of each trait, using the weights described in the *Estimate of breeding value* part. The genetic standard deviation of the synthetic breeding goal was calculated as $\sigma_{H}=\sqrt{Var(\sum_{k=1}^{6}a_{k}X_{k})}\;=\sqrt{\sum_{k=1}^{6}a_{k}^{2}\sigma_{k}^{2}+2\sum_{k<j}a_{k}a_{j}\rho_{kj}\sigma_{k}\sigma_{j}}$ where k and j denoted two traits, $X_{k}$ the TBV of the k trait, $a_{k}$ the weight of trait k in the breeding goal H, $\sigma_{k}²$ the genetic variance of trait k and $\rho_{kj}$ the genetic correlation between the k and j traits. All synthetic breeding goals were standardized using the mean and standard values from generation G₀. The results represented average ΔG across the 30 replicates. The mean annual ΔG was calculated as the cumulated ΔG divided by 11.5 years. The significance of the differences in annual ΔG between scenarios was assessed using Tukey’s test, with a p-value threshold of 0.05.

The mean cumulated ΔF was calculated as the difference between the mean level of inbreeding of the population at generation G_0_. The result was averaged across the 30 replicates to obtain the final cumulated ΔF values. The ΔF was also calculated annually by dividing the mean cumulated ΔF after 600 weeks of selection by 11.5. A Tukey test (with a 5% threshold) was used to detect significant differences in annual ΔF.

## Results

### Genetic gain and inbreeding rate

**Fig. 4:**
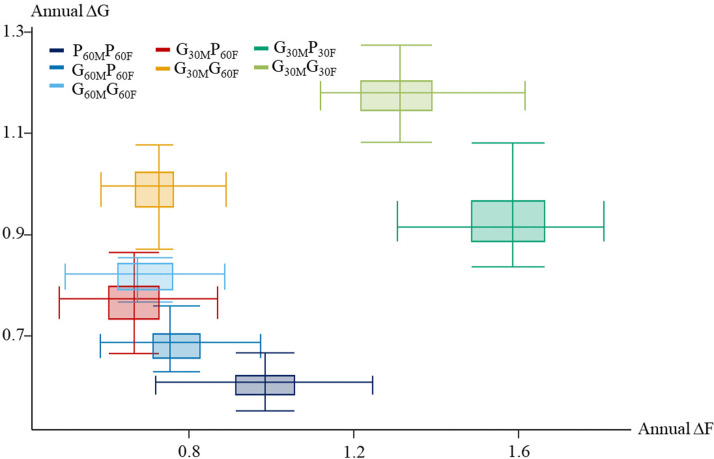
**Annual inbreeding rate (ΔF) and annual genetic gain (ΔG) for the breeding objective, expressed in standard deviation of the first generation for the 7 tested scenarios. T**he median of each scenario is indicated by the crossover point of the whiskers for ∆G and ∆F. The borders of the box indicate the 1st and 3rd quartiles for both axes (∆G and ∆F). The whiskers indicate the maximum and minimum values for each scenario.

All scenarios differed significantly in terms of mean annual genetic gain (ΔG) for the synthetic combination of the 6 traits TBVs, as calculated across the 30 replicates (Table 3). Additional Figure S2 displays the evolution of the ΔG over the 600 weeks of selection. Additional Figure S3 displays the evolution of the ΔG per generation. The PBLUP scenario P_60M_P_60F_, based solely on phenotypic and pedigree information, yielded the lowest annual ΔG (0.61 σ_A_). Including male genotypes and using ssGBLUPm (G_60M_P_60F_) increased annual ΔG by 13% compared to PBLUP, reaching 0.69 σ_A_. In terms of ΔF, the P_60M_P_60F_ scenario showed an annual inbreeding rate of 0.98%, which was reduced by 20% when male genotypes were included in the evaluations (G_60M_P_60F_, Δ*F* = 0.78%). Distribution of ΔF compared to ΔG after 600 weeks of selection were computed in Table 3 and graphically represented in Fig. 4. Additional Figure S4 displays the evolution of the ΔF over the 600 weeks of selection. Additional Figure S5 displays the evolution of the ΔF per generation.

**Table 3:** Results of mean annual genetic gain (ΔG) for the breeding objective expressed in genetic standard deviation of the G_0_ generation and annual mean increase in inbreeding rate (ΔF) expressed in percentage (%) for the seven simulated scenarios. *L*: generation interval, BLUP: type of BLUP model used in the evaluations.

*Scenario*	*L*	*BLUP*	*ΔG*	*ΔF*
P_60M_P_60F_	60	PBLUP	0.61	0.98
G_60M_P_60F_	60	ssGBLUPm	0.69	0.78
G_60M_G_60F_	60	ssGBLUPmf	0.82	0.69
G_30M_P_60F_	45	ssGBLUPm	0.77	0.67
G_30M_G_60F_	45	ssGBLUPmf	0.99	0.72
G_30M_P_30F_	30	ssGBLUPm	0.93	1.56
G_30M_G_30F_	30	ssGBLUPmf	1.17	1.29

The ssGBLUPmf scenarios consistently achieved higher annual ΔG than their ssGBLUPm counterparts (Table 3). The inclusion of female genotypes led to a higher ΔG for reduced *L* scenarios*,* with a +26% increase for 30-week *L* (G_30M_G_30F_ vs G_30M_P_30F_) and +28% for 45-week *L* (G_30M_G_60F_ vs G_30M_P_60F_) compared to +19% for 60-week *L* (G_60M_G_60F_ vs G_60M_P_60F_). The highest annual ΔG (1.17σ_A_) was obtained with the 30-week *L* scenario G_30M_G_30F_, with discrete generations and where both sexes had genotypes. The impact of female genotyping on ΔF was not significant for 60-week or 45-week *L* (p-values between 0.93 and 0.58), and only moderately lower for 30-week *L* (−17%).

Reducing *L* to 30 weeks for both sexes in discrete generation scenarios increased ΔG by +43% under ssGBLUPmf (G_30M_G_30F_ vs G_60M_G_60F_) and by +35% under ssGBLUPm (G_30M_P_30F_ vs G_60M_P_60F_). This improvement in genetic gain was accompanied by higher ΔF, but the increase in ΔF was less pronounced with ssGBLUPmf (+87%) than with ssGBLUPm (+100%), indicating more desirable values of annual ΔG and ΔF when using ssGBLUPmf.

### Prediction accuracy

Mean prediction accuracies across the 30 replicates and over 600 weeks of selection are reported in Table 4 and their distribution is shown in Additional Figure S6. Globally, prediction accuracy was always higher for 60-week traits than for 90-week traits, regardless of the scenario or sex of the candidates. In most scenarios (P_60M_P_60F_, G_60M_G_60F_, G_30M_P_60F_, G_30M_G_60F_), prediction accuracies were higher for females than for males. In contrast, in scenario G_30M_P_30F_, males consistently showed higher prediction accuracy than females. Scenario G_30M_G_30F_ showed balanced prediction accuracies between sexes, while G_60M_P_60F_ resulted in higher prediction accuracy for females for 60-week traits and for males for 90-week traits.

**Table 4:** Prediction accuracy for the 7 tested scenarios, averaged across the 30 replicates and 600 weeks of selection, distinctly for 60-week traits (EW_60_, ESS_60_ and LR_60_) and for 90-week traits (EW_90_, ESS_90_, LR_90_), for males, for females and for both sexes (population). Within each line, two values of prediction accuracy sharing the same symbol (*, #, or †) were not significantly different from each other.

*Scenarios*	*Sex*	*P_60M_P_60F_*	*G_60M_P_60F_*	*G_60M_G_60F_*	*G_30M_P_60F_*	*G_30M_G_60F_*	*G_30M_P_30F_*	*G_30M_G_30F_*
**60-week traits**	M	0.56^#^	0.68	0.80	0.56^#^	0.74	0.55	0.66
	F	0.70*	0.70*	0.83	0.70*	0.82	0.46	0.66
	population	0.63*	0.69	0.81	0.63*	0.78	0.51	0.66
**90-week traits**	M	0.48	0.60	0.71	0.52	0.67	0.46	0.58
	F	0.56*	0.57	0.72	0.56*	0.70	0.39	0.58
	population	0.52	0.59*	0.72	0.54	0.68	0.42	0.58
**All traits**	M	0.52	0.64	0.75	0.54	0.70	0.51	0.62
	F	0.63*^#^	0.64^†^	0.77	0.63*^†^	0.76	0.42	0.62^#^
	population	0.58	0.64	0.76	0.58	0.73	0.47	0.62

Including male genotypes in the evaluations of scenarios with a 60-week *L* and discrete generations improved the prediction accuracy of evaluation which reached 0.64 for both sexes (Table 4). Switching from ssGBLUPm to ssGBLUPmf improved general prediction accuracy across all types of scenarios: +19% for 60-week*L* (G_60M_G_60F_ vs G_60M_P_60F_), +26% for 45-week *L* (G_30M_G_60F_ vs G_30M_P_60F_), and +32% for 30-week*L* (G_30M_G_30F_ vs G_30M_P_30F_). Including female genotypes particularly improved female prediction accuracy, especially in discrete generation scenarios with reduced *L, with increases of* +20% for 60-week *L* (G_60M_G_60F_ vs G_60M_P_60F_) and up to +48% for 30-week *L* (G_30M_G_30F_ vs G_30M_P_30F_). Male prediction accuracy also improved under ssGBLUPmf, with larger increases in overlapping generation scenarios (G_30M_G_60F_ vs G_30M_P_60F_, +30%) than in discrete ones (∼+20%). ssGBLUPmf also reduced the discrepancy between male and female prediction accuracies compared to ssGBLUPm for all the types of scenarios. The increase in prediction accuracy from including female genotypes was greater for 90-week traits than for 60-week traits under the 30-week *L* scenarios (G_30M_G_30F_ vs G_30M_P_30F_), with increases of +29% and +38%, respectively. A smaller difference was observed in the overlapping generation scenario (G_30M_G_60F_ vs G_30M_P_60F_, +23% for 60-week traits and +26% for 90-week traits). In contrast, under the 60-week *L* scenarios (G_60M_G_60F_ vs G_60M_P_60F_), the gain was similar for both trait types (+20%).

### Overlapping generation scenarios

Overlapping generations offered a compromise between high annual ΔG and controlled annual ΔF (Table 3). The ssGBLUPmf scenario G_30M_G_60F_ achieved intermediate annual ΔG (0.99 σ_A_) between the discrete 30*-*week *L* (G_30M_G_30F_) and 60-week*L* (G_60M_G_60F_), representing an improvement of +21% compared to G_60M_G_60F_. Similarly, the ssGBLUPm overlapping scenario G_30M_P_60F_ showed a +12% increase in annual ΔG compared to G_60M_P_60F_ and was only 6% lower than G_60M_G_60F_. Full genomic ssGBLUPmf scenario with overlapping generations G_30M_G_60F_ achieved intermediate annual ΔG between 30-week *L* scenarios G_30M_P_30F_ (+6%) and G_30M_G_30F_ (−15%) with discrete generations. The ssGBLUPm scenario G_30M_P_60F_ achieved intermediate ΔG between 60-week *L* scenarios G_60M_G_60F_ (-6%) and in G_60M_P_60F_ (+12%).

Prediction accuracy in overlapping (Table 4) G_30M_G_60F_ systematically reached higher prediction accuracies than scenarios with a 30-week *L* G_30M_P_30F_ (+55%) and G_30M_G_30F_ (+18%). Prediction accuracy in G_30M_P_60F_ was lower (−24%) than in G_60M_G_60F_ and in G_60M_P_60F_ (−14%). The prediction accuracies of the female evaluations in scenarios with overlapping generation were not significantly different than the prediction accuracies observed for scenarios with a 60-week *L* under ssGBLUPm (G_30M_P_60F_ vs G_60M_P_60F_) and 2% higher under ssGBLUPmf (G_30M_G_60F_ vs G_60M_G_60F_). The male prediction accuracies were lower compared to scenarios with a 60-week *L* and discrete generations by -16% for ssGBLUPm (G_30M_P_60F_ vs G_60M_P_60F_) and −7% for ssGBLUPmf scenario (G_30M_G_60F_ vs G_60M_G_60F_).

Overlapping generations scenarios achieved less annual ΔF than scenarios with discrete generations and a 30-week *L*: −55% for G_30M_P_60F_ vs G_30M_P_30F_ and −44% for G_30M_G_60F_ vs G_30M_G_30F_. Compared to scenarios with a conventional 60-week *L*, overlapping generation scenarios achieved slightly higher (+14%) annual ΔF under ssGBLUPm (G_30M_P_60F_ vs G_60M_P_60F_) and similar annual ΔF under ssGBLUPmf (G_30M_G_60F_ vs G_60M_G_60F_).

## Discussion

In this study, various selection strategies were simulated to understand the trade-offs between reducing *L* and implementing genomic selection (GS) in selection programs aimed at extending laying period to 90 weeks. Three types of scenarios were considered: PBLUP with a 60-week *L* in discrete generations, ssGBLUPm scenarios including male genotypes only, and ssGBLUPmf scenarios with complete genotyping of males and females. For both ssGBLUPm and ssGBLUPmf, three *L* strategies were tested to assess the impact of GS in different selection schemes: discrete generations with a conventional 60-week *L* or a reduced 30-week *L*, and overlapping generations with 45-week *L*, where males were selected at 30 weeks and females at 60 weeks.

The prediction accuracy and inbreeding trends observed here were consistent with previous findings (Pocrnic et al., 2023; Büttgen et al., 2025), and the ΔG values were also comparable to those reported in similar contexts. Beyond confirming these earlier results, this study provides new insights into the potential of genomic selection to extend laying periods for both production and quality traits. These findings are particularly relevant in a context where population-scale genotyping is becoming increasingly feasible due to advances in next-generation sequencing technologies.

### Limits of the simulations

Generation intervals were defined as multiples of 30 weeks, even though the minimum age for hens to produce hatchable eggs is generally around 32 weeks (Mróz et al., 2022). Nevertheless, sexual maturity is under selection, and several simulation studies suggested to reduce *L* to half a year (Wolc et al., 2011b; Sitzenstock et al., 2013).

The utilization of real genotypes in genome simulations, as opposed to entirely simulated ones, is a subject of debate due to the exclusion of certain micro-chromosomes from the simulations, as they are not all represented on SNP chips. However, in comparison to the use of fully simulated genomes, which permit the inclusion of these chromosomes, initiating the simulation with real genomic data allows for a more effective accounting of the linkage disequilibrium structure. This structure is not linear between chromosomes in avian species (Groenen et al., 2009). Additionally, all genotyped individuals used to simulate the population were assumed to be unrelated, which is not entirely accurate an can introduce bias. This assumption carries the risk of inbreeding progressing more rapidly than it would with entirely simulated founder populations. Despite these drawbacks, the results were comparable to those of studies in which genomes were entirely simulated (Pocrnic et al., 2023; Büttgen et al., 2025).

The current study does not consider the impact of **inbreeding depression (ID)** on genetic gain as traits and underlying QTLs were simulated to be purely additive. Inbreeding can lead to the emergence of deleterious recessive alleles, which can indirectly affect genetic progress and the available genetic variability. Previous research has demonstrated that ID influences genetic gain for traits such as egg number and sexual maturity in layers (Sewalem et al., 1999). Recommendations about minimum effective population size were published in the 80′s (Franklin et al., 1980; Soulé 1980) and reviewed in 2014 (Frankham et al., 2014) to avoid ID. However, due to the cross-breeding steps inherent in poultry breeding schemes, the economic consequences of ID are less severe compared to other species (Leroy 2014). The simulations conducted in this study span 600 weeks of selection, resulting in nearly constant genetic gain. If extended over a longer period, this gain would likely reach a ceiling, particularly with reduced genetic variability. Although the current simulations did not incorporate inbreeding management tools such as **Optimal Contribution Selection (OCS)**, it is recommended to integrate these tools into the selection criteria to mitigate the effects of ID and sustain genetic progress (Meuwissen, 1997). The interest of OCS has already been demonstrated for laying hens breeding in multiple studies (Pocrnic et al., 2023; Büttgen et al., 2025).

### Genomic selection in females

Incorporating female genotypes increased evaluation accuracy for both sexes, across all scenarios. This led to improved annual ΔG, confirming earlier results (Wolc et al., 2011b). However, this benefit was more pronounced when candidates were distantly related to phenotyped individuals, e.g., for 90-week traits in 60-week *L* scenarios, or for all traits when *L* was reduced to 30 weeks. These results complemented those of Picard Druet et al. (2020), highlighting the necessity to use GS in a context where selection must be made before trait phenotyping, such as persistency traits.

### Reduction of generation intervals

Reducing L from 60 to 30 weeks decreased accuracy, as expected. This decline was likely due to an increased dependence on phenotypic information from earlier generations. It is well established that genomic prediction accuracy declines as the genetic distance between reference and candidate populations increases (Habier et al., 2010; Pszczola et al., 2012b). Moreover, fewer contemporaries were phenotyped in 30-week L scenarios (2000 vs. 3000), further contributing to the drop in accuracy (Yang and Su, 2016). These elements explain the lower accuracies observed for 90-week traits compared to 60-week traits, across all scenarios. The inclusion of female genotypes partially offset this drop, particularly for 90-week traits. The strongest positive impact of female genotyping was observed under the 30-week *L* scenario for 90-week traits, when the generational gap between candidate females and phenotyped females was the highest of all scenarios. This supports the idea that GS is especially valuable when traits are recorded after selection and females don’t benefit from their own performances or performances of close relatives in the evaluation.

Combining a reduced *L* with full population genotyping achieved the highest annual ΔG, as the loss in accuracy was compensated by increased generational turnover. However, this approach also led to the highest annual ΔF. When expressed per generation, ΔF remained similar across scenarios, confirming that the observed annual increase was mainly driven by faster generational turnover rather than by a misestimating of the relationship matrix or a failure to capture family structure accurately (Hayes et al., 2009). While this strategy maximizes ΔG, it also raises concerns about sustainability. In the case of scenarios with a 30-week *L*, the inbreeding rate were relatively high compared to FAO recommendations for sustainable breeding programs (FAO, 2010). It recommends that the inbreeding rate should be maintained below the range of 0.5 to 1% per year to avoid risks of genetic disorders and ID. In 2013, these recommendations were reviewed for commercial breeds in 2013 (Boettcher et al., 2013). A consensual threshold of 2% of maximum increase in inbreeding rate per generation was admitted.

### Overlapping generations

Schemes involving overlapping generations consistently demonstrated superior performance compared to discrete generation strategies in terms of annual ΔG. In these overlapping generation scenarios, 3000 males and females were born every 30-weeks *L* of 30 weeks for males. However, the actual *L* extended to 45 weeks because females were 60 weeks old when mated with males. Consequently, these scenarios involved 9000 individuals when calculated per generation.

The selection intensity remained consistent across all scenarios, but the total number of contributors per generation varied. Specifically, the number of males and females was maintained at constant levels between discrete and overlapping generation scenarios. After 600 weeks of selection, 1575 male and 12 600 female breeders have been selected in 45 and 30-week *L* scenarios. It means that, in overlapping generation scenarios with a 45-weeks *L*, 118 males and 945 females contributed to each generation, compared to 75 males and 600 females in discrete generation scenarios. As a result, the annual ΔF was logically lower in overlapping generation scenarios (Bijma et al., 2001).

Although comparing this strategy to other scenarios is unconventional due to differing numbers of candidates per generation, the practical implementation of such breeding programs closely resembles those with discrete generations and a 30-week generation interval. This similarity makes it feasible to consider overlapping generation strategies for layer breeding programs.

This strategy of overlapping scenario, even if really different by the number of contributors, provided a compromise between the ΔG of 30-week *L* and the accuracy of 60-week *L*. Since female phenotypes remained available, female accuracy was not affected. Male candidates, however, depended more on ancestral information, slightly lowering their evaluation accuracy. Yet, the increased generational turnover outweighed this effect, leading to improved annual ΔG.

This study demonstrated that shortening generation interval for both males and females maximized annual genetic gain, driven by faster generational turnover, but at the cost of lower accuracy and higher annual inbreeding rate. Including female genotypes improved accuracy, especially for late-measured 90-week traits, and partially compensated for the loss of information due to reduce generation interval. This highlights the relevance of genomic selection when selecting for late-expressed traits in generation interval reduced breeding programs. Fine inbreeding management methods enabled by genomic selection should also be tested to optimize these scenarios. Overlapping generation strategies—reducing generation interval only on the male side—offered a good compromise, maintaining intermediate annual genetic gain while limiting annual inbreeding rate through fewer generations per year and more contributors per generation. However, the implementation of such strategies must consider practical constraints like housing capacity and economic considerations.

## Ethics approval and consent to participate

All blood samples were carried out as part of the commercial and selection activities of Novogen. These animals studied and the scientific investigations described herein are therefore not to be considered as experimental animals per se, as defined in EU directive 2010/63 and subsequent national application texts. Consequently, we did not seek ethical review and approval of this study as regarding the use of experimental animals. All animals were reared in compliance with national regulations pertaining to livestock production and according to procedures approved by the French Veterinary Services.

## Funding

This work was supported by the French Institut National de Recherche pour l'agriculture, l'alimentation et l'environnement (INRAE) and L’institut Agro Rennes-Angers [recipient Mathilde Doublet]

## CRediT authorship contribution statement

**Mathilde Doublet:** Conceptualization, Investigation, Methodology, Writing – original draft, Writing – review & editing. **Frédéric Lecerf:** Conceptualization, Funding acquisition, Project administration, Supervision, Writing – review & editing. **Torsten Pook:** Conceptualization, Investigation, Methodology, Software, Supervision, Validation, Writing – review & editing. **Hervé Chapuis:** Conceptualization, Investigation, Validation, Writing – review & editing. **Jérôme Raoul:** Conceptualization, Methodology, Validation, Writing – review & editing. **Florian Herry:** Conceptualization, Methodology, Software, Validation, Writing – review & editing. **Sophie Brad-Fudulea:** Conceptualization, Data curation, Resources, Validation, Writing – review & editing. **Gwendal Restoux:** Conceptualization, Methodology, Validation, Writing – review & editing. **Florence Phocas:** Conceptualization, Methodology, Validation, Writing – review & editing. **Sophie Allais:** Conceptualization, Funding acquisition, Investigation, Methodology, Supervision, Validation, Writing – review & editing.

## Disclosures

The authors declare that they have no known competing financial interests or personal relationships that could have appeared to influence the work reported in this paper.

## Appendix Supplementary materials

Image, application 1

Image, application 2

Image, application 3

Image, application 4

Image, application 5

Image, application 6

## Footnotes

Supplementary material associated with this article can be found, in the online version, at doi:10.1016/j.psj.2026.106870.
