# Supplementary figures and images for: Stochastic simulations to optimize genomic selection for laying hens: Impact of generation interval and genotyping in the context of extended laying period
Source: Poult Sci. 2026 Mar 27;105(7):106870. doi: 10.1016/j.psj.2026.106870 (PMC13126499; doi:10.1016/j.psj.2026.106870)

**Additional Figure S1:** Description of the 7 tested breeding programs.

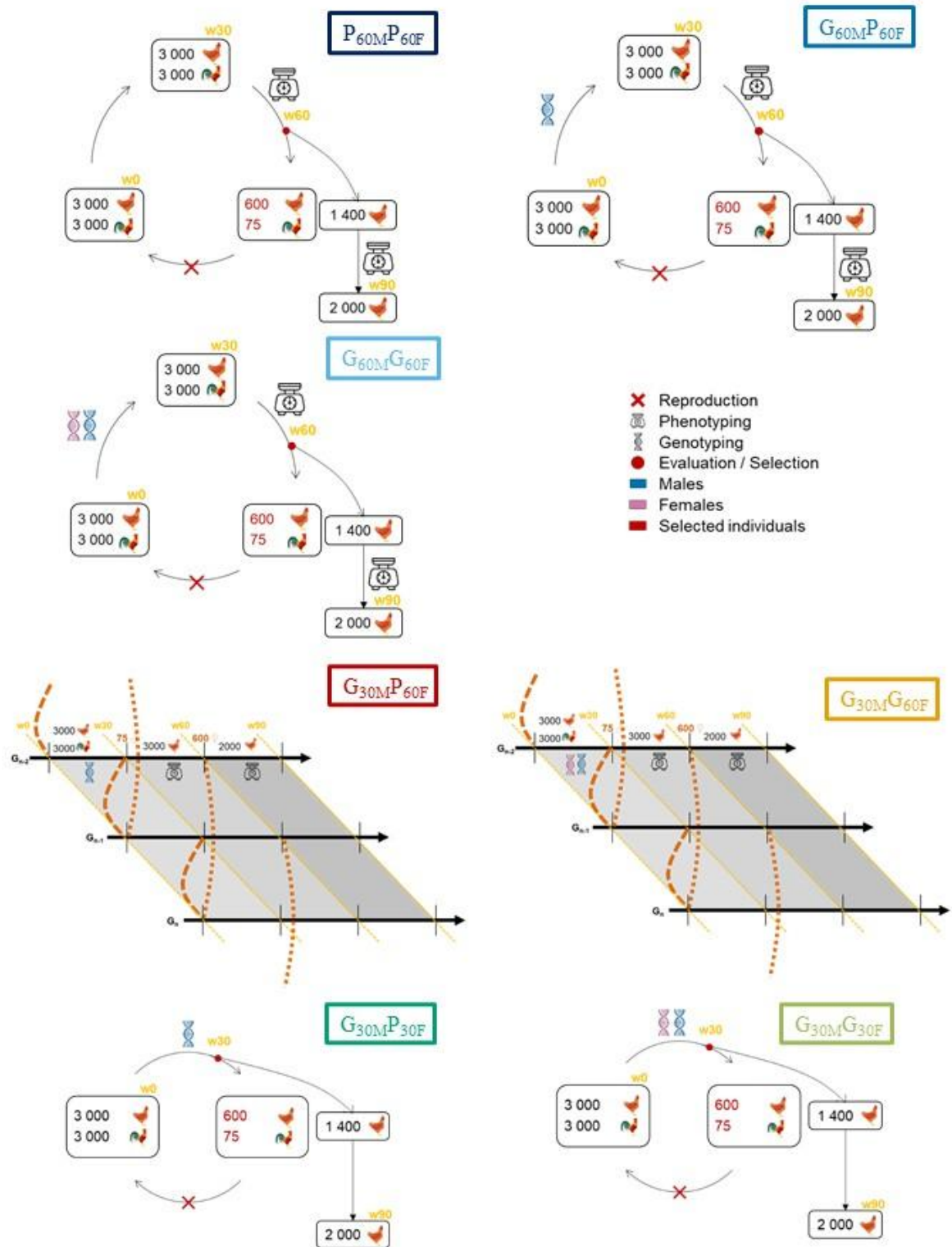

Supplement: Supplementary file 1 [file mmc1.pdf]
