# Supplementary material for: Stochastic simulations to optimize genomic selection for laying hens: Impact of generation interval and genotyping in the context of extended laying period
Source: Poult Sci. 2026 Mar 27;105(7):106870. doi: 10.1016/j.psj.2026.106870 (PMC13126499; doi:10.1016/j.psj.2026.106870)

Additional Figure S2: Mean genetic gain (ΔG) per year for the synthetic combination of TBVs calculated as $0.06 * TBV_{EW_{60}} + 0.16 * TBV_{ESS_{60}} + 0.22 * TBV_{LR_{60}} + 0.06 * TBV_{EW_{90}} + 0.22 * TBV_{ESS_{90}} + 0.28 * TBV_{LR_{90}}$. Description: 95% confidence intervals are given for the average value and based on 30 independent replicates each. ΔG are expressed in genetic standard deviation of the G_0 generation

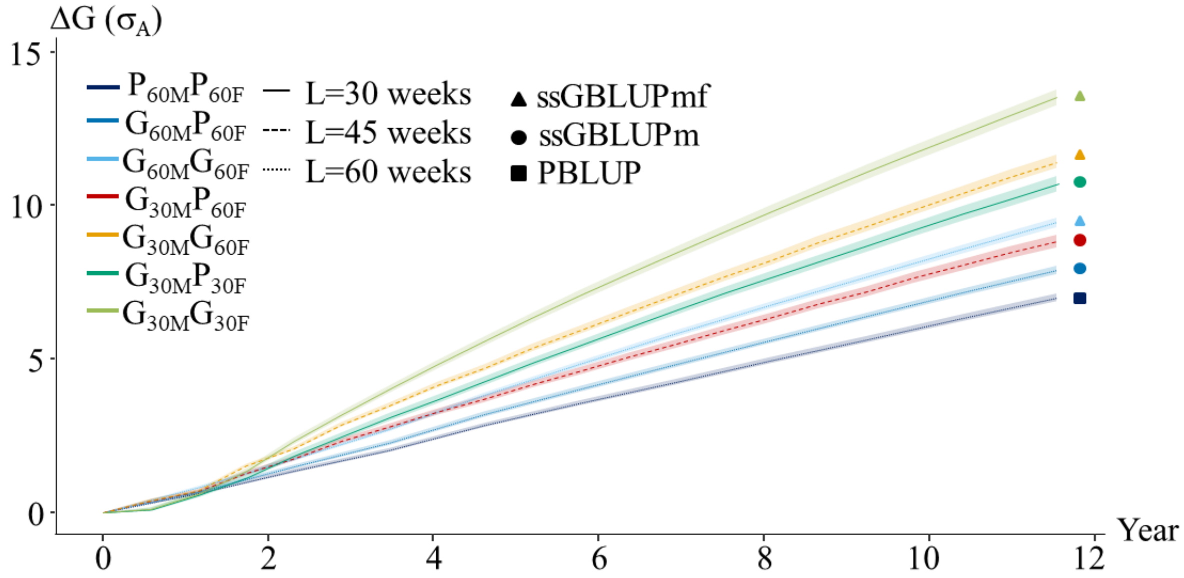

Supplement: Supplementary file 2 [file mmc2.pdf]
