# Supplementary material for: Stochastic simulations to optimize genomic selection for laying hens: Impact of generation interval and genotyping in the context of extended laying period
Source: Poult Sci. 2026 Mar 27;105(7):106870. doi: 10.1016/j.psj.2026.106870 (PMC13126499; doi:10.1016/j.psj.2026.106870)

Additional Figure S5: Inbreeding coefficient development for 10 generations according to the 7 tested scenarios having different generation intervals (*L*). Description: 95% confidence intervals are given for the average value and based on 30 independent replicates each.

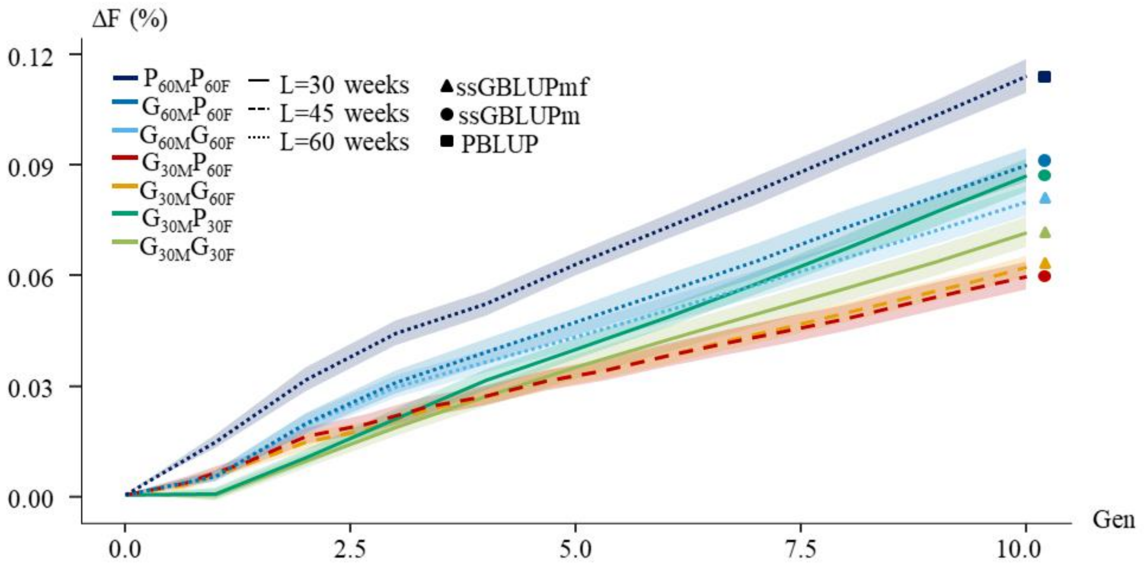

Supplement: Supplementary file 5 [file mmc5.pdf]
