# Supplementary material for: Stochastic simulations to optimize genomic selection for laying hens: Impact of generation interval and genotyping in the context of extended laying period
Source: Poult Sci. 2026 Mar 27;105(7):106870. doi: 10.1016/j.psj.2026.106870 (PMC13126499; doi:10.1016/j.psj.2026.106870)

Additional Figure S6: Distributions of the mean accuracy of evaluation across the 600 weeks. Egg Weight (EW_{60}), Egg Shell Strength (ESS_{60}), Laying Rate (LR_{60}) measured at 60 weeks and for Egg Weight (EW_{90}), Egg Shell Strength (ESS_{90}), Laying rate (LR_{90}) measured at 90 weeks, distinctly for males (M) and females (F) or at a population level for the 7 scenario

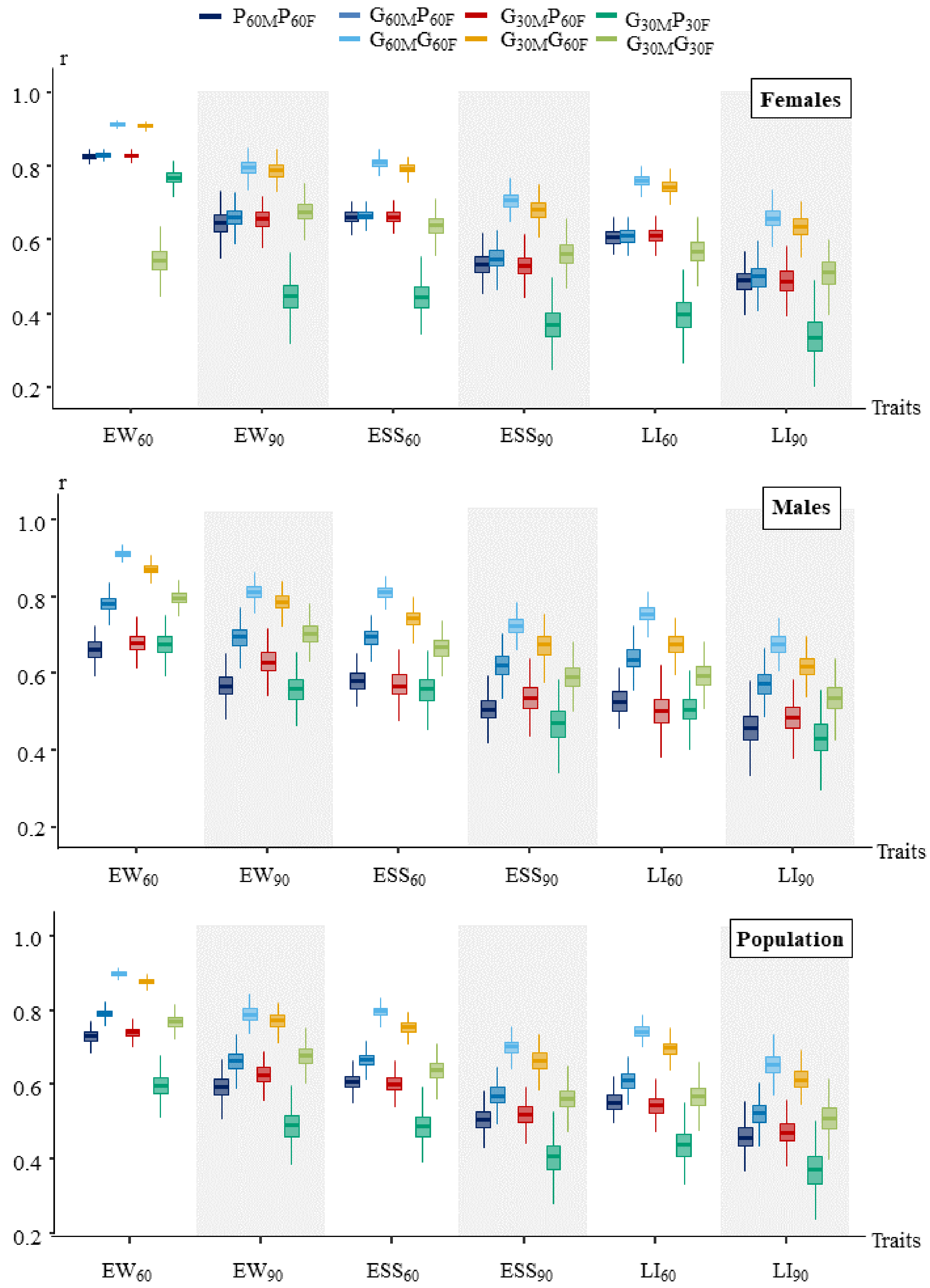

Supplement: Supplementary file 6 [file mmc6.pdf]
